# Supplementary material for: Causal Relationship between Aging and Anorexia Nervosa: A White-Matter-Microstructure-Mediated Mendelian Randomization Analysis
Source: Biomedicines. 2024 Aug 16;12(8):1874. doi: 10.3390/biomedicines12081874 (PMC11351342; doi:10.3390/biomedicines12081874)
Supplement: Supplementary file 1 [file biomedicines-12-01874-s001.zip › Supplementary Figure/Figure S4/Singe SNP plot of MR analysis for TL to ubm-b-1524.pdf]

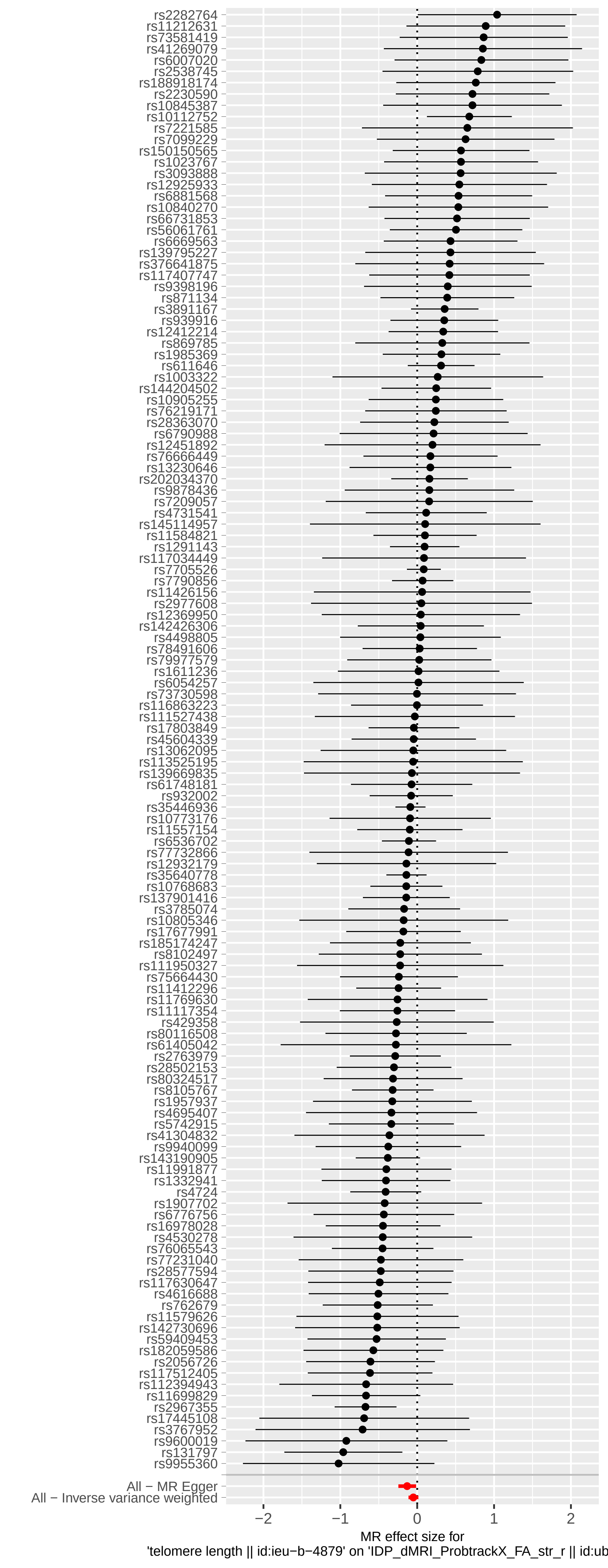
rs2282764
rs11212631
rs73581419
rs41269079
rs6007020
rs2538745
rs188918174
rs2230590
rs10845387
rs10112752
rs7221585
rs7099229
rs150150565
rs1023767
rs3093888
rs12925933
rs6881568
rs10840270
rs66731853
rs56061761
rs6669563
rs139795227
rs376641875
rs117407747
rs9398196
rs871134
rs3891167
rs939916
rs12412214
rs869785
rs1985369
rs611646
rs1003322
rs144204502
rs10905255
rs76219171
rs28363070
rs6790988
rs12451892
rs76666449
rs13230646
rs202034370
rs9878436
rs7209057
rs4731541
rs145114957
rs11584821
rs1291143
rs117034449
rs7705526
rs7790856
rs11426156
rs2977608
rs12369950
rs142426306
rs4498805
rs78491606
rs79977579
rs1611236
rs6054257
rs73730598
rs116863223
rs111527438
rs17803849
rs45604339
rs13062095
rs113525195
rs139669835
rs61748181
rs932002
rs35446936
rs10773176
rs11557154
rs6536702
rs77732866
rs12932179
rs35640778
rs10768683
rs137901416
rs3785074
rs10805346
rs17677991
rs185174247
rs8102497
rs111950327
rs75664430
rs11412296
rs11769630
rs11117354
rs429358
rs80116508
rs61405042
rs2763979
rs28502153
rs80324517
rs8105767
rs1957937
rs4695407
rs5742915
rs41304832
rs9940099
rs143190905
rs11991877
rs1332941
rs4724
rs1907702
rs6776756
rs16978028
rs4530278
rs76065543
rs77231040
rs28577594
rs117630647
rs4616688
rs762679
rs11579626
rs142730696
rs59409453
rs182059586
rs2056726
rs117512405
rs112394943
rs11699829
rs2967355
rs17445108
rs3767952
rs9600019
rs131797
rs9955360
All − MR Egger
All − Inverse variance weighted
−2
−1
0
1
2
MR effect size for
'telomere length || id:ieu−b−4879' on 'IDP_dMRI_ProbtrackX_FA_str_r || id:ub
